# Reactive molecular dynamics simulation of the high-temperature pyrolysis of 2,2′,2′′,4,4′,4′′,6,6′,6′′-nonanitro-1,1′:3′,1′′-terphenyl (NONA)

**DOI:** 10.1039/c9ra10261b

**Published:** 2020-02-04

**Authors:** Liang Song, Feng-Qi Zhao, Si-Yu Xu, Xue-Hai Ju

**Affiliations:** Key Laboratory of Soft Chemistry and Functional Materials of MOE, School of Chemical Engineering, Nanjing University of Science and Technology Nanjing 210094 P. R. China xhju@njust.edu.cn; Laboratory of Science and Technology on Combustion and Explosion, Xi'an Modern Chemistry Research Institute Xi'an 710065 P. R. China

## Abstract

2,2′,2′′,4,4′,4′′,6,6′,6′′-Nonanitro-1,1′:3′,1′′-terphenyl (NONA) is currently recognized as an excellent heat-resistant explosive. To improve the atomistic understanding of the thermal decomposition paths of NONA, we performed a series of reactive force field (ReaxFF) molecular dynamics simulations under extreme conditions of temperature and pressure. The results show that two distinct initial decomposition mechanisms are the homolytic cleavage of the C–NO_2_ bond and nitro–nitrite (NO_2_ → ONO) isomerization followed by NO fission. Bimolecular and fused ring compounds are found in the subsequent decomposition of NONA. The product identification analysis under finite time steps showed that the gaseous products are CO_2_, N_2_, and H_2_O. The amount of CO_2_ is energetically more favorable for the system at high temperature or low density. The carbon-containing clusters are a favorable growth pathway at low temperatures, and this process was further demonstrated by the analysis of diffusion coefficients. The increase of the crystal density accelerates the decomposition of NONA judged by the analysis of reaction kinetic parameters and activation barriers. In the endothermic and exothermic stages, a 20% increase in NONA density increases the activation energies by 3.24 and 0.48 kcal mol^−1^, respectively. The values of activation energies (49.34–49.82 kcal mol^−1^) agree with the experimental data in the initial decomposition stage.

## Abstract 2

The bimolecular and fused ring compounds are found in the high-temperature pyrolysis of NONA using ReaxFF molecular dynamics simulations.

## Graphical abstract

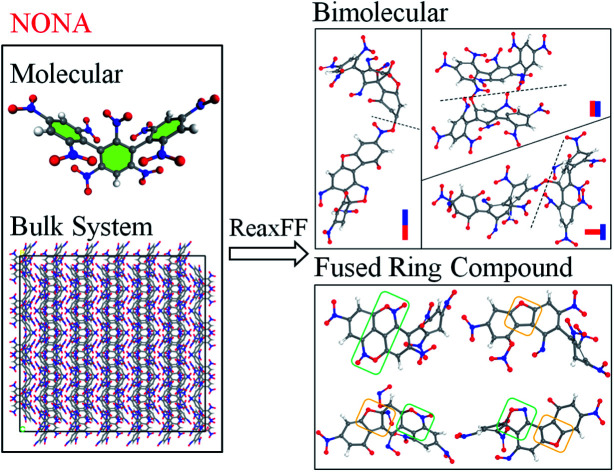

## 1. Introduction

Increases in the depth of oil and gas wells have led to corresponding changes in drilling and operating conditions. For the explosives employed, the increase in the temperature and pressure during production operations and trouble-shooting is of decisive significance. In particular, super heat-resistant energetic materials maintain stability at temperatures of up to 250 °C.^1–3^ 2,2′,2′′,4,4′,4′′,6,6′,6′′-Nonanitro-1,1′:3′,1′′-terphenyl (NONA, C_18_H_5_O_18_N_9_) that is safe, reliable and stable at high temperature is currently recognized as a promising heat-resistant explosive (decomposition temperature: 440–450 °C).^4–6^ NONA, as one of the organic polynitro compounds, releases large amounts of energy during thermal decomposition. Hence, it plays a crucial role in the exploration and exploitation of oil or space. Understanding the physical and chemical events is essential to obtain modified models for its combustion and detonation. However, the evolution details of the ignition and explosion of the condensed phase NONA remain unclear. The rapid reaction process of energetic materials under extreme conditions involves short time scales and complex chemical reaction kinetics, which poses significant challenges to current experimental techniques. Reactive molecular dynamics (ReaxFF MD) simulations enable to directly probe the evolution processes of chemical reaction under the microscopic scale. The ReaxFF MD simulations have been widely used to explore the thermal decomposition mechanism of condensed phase explosives, such as nitromethane (NM),^7^ TNT,^8,9^ RDX,^10^ HMX,^11^ CL-20,^12^ and 1,3,5-triamino-2,4,6-trinitrobenzene (TATB).^13^ Nitroaromatic explosives have higher activation energies in the condensed phase as can be seen in Table 1, and the major final products are N_2_, CO_2_, and H_2_O.

**Table 1:** Arrhenius thermolysis parameters and major products of nitroaromatic and nitramine explosives by using ReaxFF MD simulation

Molecules	Reaction step	Pre-exponential factor, ln *A* (s^−1^)	Activation energy, *E*_a_ (kcal mol^−1^)	Major final products
TNT^9^	1	33.6	35.0	N_2_, CO_2_, H_2_O, CO
	2	30.0	27.8	
TATB^13^	1	33.2	30.2	N_2_, CO_2_, H_2_O, CO
	2	31.0	29.7	
HNS^14^	1	6.0	37.13	N_2_, H_2_O, CO_2_, H_2_
	2	28.8	24.76	
RDX^10^	1	—	23–26.6	N_2_, CO_2_, H_2_O, CO
HMX^11^	1	33.8	25.1	N_2_, CO_2_, H_2_O, CO
	2	29.3	22.6	
CL-20 (ref. 12)	1	—	30.6	N_2_, H_2_O, CO_2_, H_2_

Considering that NONA belongs to nitro-based organic explosives, we summarize the basic research and applied research on the thermal decomposition of a literature review. Rom *et al.*^7^ used ReaxFF MD simulations to investigate the decomposition mechanism of hot liquid NM at various compressions. They observed two different initial thermal decomposition schemes: (1) unimolecular C–N bond cleavage followed by subsequent C–NO_2_ steps forming eventually three NO_2_ (g) molecules and ·CH_3_ radical at low density; (2) the formation of CH_3_NO *via* H-transfer and N–O bond rupture at high density. Rom *et al.*^8^ used ReaxFF MD simulations to analyze the thermal decomposition of liquid phase TNT at high temperature. The dissociation of the C–NO_2_ bond is putatively the initial mechanism of liquid phase TNT decomposition at low density, whereas dimer formation and decomposition at higher compressions. Furman *et al.*^9^ elucidated the difference between activation energies in gas phase and condensed phase of TNT, and discussed the uni- and bimolecular reaction mechanism of thermal decomposition of condensed phase TNT. Zhang *et al.*^13^ used the ReaxFF MD simulation to study the process of thermal decomposition of TATB to form carbon clusters. They found that TATB decomposes to form a large amount of carbon clusters (15–30% of the total mass of the system). Chen *et al.*^14^ suggested two distinct mechanisms for initial decomposition of 2,2′,4,4′,6,6′-hexanitrostilbene (HNS): C–NO_2_ bond dissociation and nitro–nitrite isomerization. The activation energy of thermolysis for NONA is a crucial parameter of performance. Zeman *et al.*^5^ have shown the activation energy for thermal decomposition of NONA (56.48 kcal mol^−1^) by the Russian manometric method. Keshavarz *et al.*^15^ predicted the activation energy of the thermolysis in the condensed state for polynitro arenes, and they obtained activation energy of NONA (51.14 kcal mol^−1^).

However, the detailed understanding of the transient evolution of condensed phase NONA as well as its interplay with the environmental conditions at initial stage of thermal decomposition is currently lacking. In this work, we performed the simulations to study the thermal decomposition of NONA. This paper is organized as follows: Computational details for both the simulation method and kinetic parameter analysis are described in Section 2. Results and discussions (decomposition pathways, exothermic phase, fragments analysis, and reaction kinetic parameters) are presented in Section 3. Finally, conclusions are summarized in Section 4.

## 2. Computational details

### 2.1 Simulation method

**Fig. 1:**
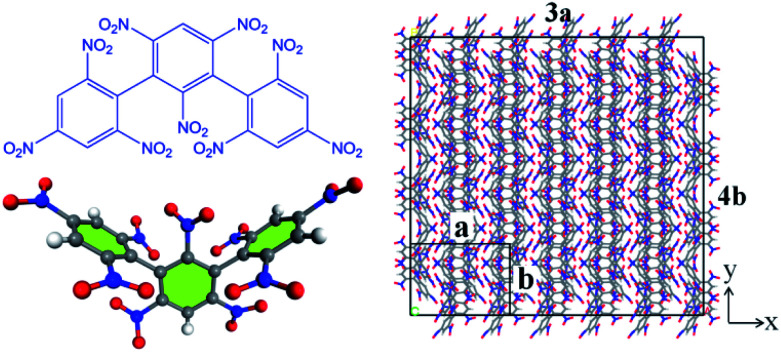
The molecular formula and three-dimensional structure of NONA (left) and structures of the NONA supercell (right) (3a × 4b × 2c).

All the molecular simulations were performed with the ReaxFF-lg force field using LAMMPS program package. The original unit cell obtained from the experiment was expanded into a 3 × 4 × 2 NONA periodic supercell, and the system contains 192 molecules (24 units, 9600 atoms).^6^ The molecular formula, three-dimensional structure and initial supercell of NONA are shown in Fig. 1. First, in order to minimize the energy, a conjugate gradient algorithm was used to relax the NONA supercell. The canonical ensemble (NVT) and the Berendsen thermostat were applied to the MD simulation with a total time of 10 ps at 300 K, which further relaxed the NONA supercell. The relaxed supercell is performed with isothermal-isobaric (NPT) MD simulation at 0 atm and 300 K for 20 ps, and the equilibrium structure was obtained. In order to verify the feasibility of the parameters, the equilibrium supercell structure was compared to the single crystal diffraction data. Then, the relaxed supercell (*V*_0_ = 4.646 × 10^3^ cm^3^, crystal density *ρ* = 1.816 g cm^−3^) was used to generate the volumetrically expanded and compressed supercells and would be used in a high temperature pyrolysis reaction. By changing the lattice parameters of the fixed fractional coordinates of the superelement, we obtained the corresponding 1.1*V*_0_ (10% expansion, *ρ* = 1.365 g cm^−3^) and 0.9*V*_0_ (10% compression, *ρ* = 2.492 g cm^−3^) supercells. To further reduce any artificial pressure caused by altering unit cell parameters, the supercell is minimized by energy and further evolved by the microcanonical ensemble (NVE) for another 5 ps to obtain a fully relaxed supercell for thermal decomposition simulation. Then, the relaxed supercell was used as the initial structure for subsequent MD simulations. Then, ReaxFF-lg isothermal–isochoric MD (NVT-MD) simulations were performed for 200 ps at 1800, 2250, 2500, 3000 and 3500 K, respectively, controlled by the Berendsen thermostat. The damping constant was 100.0 fs. Periodic boundary conditions are applied to all simulations. Newton's equation of motion was calculated by using the velocity–Verlet algorithm with a time step of 0.1 fs. An analysis of the fragments was performed with a 0.3 bond order cutoff value for each atom pairs to identify chemical species. The information of dynamic trajectory was recorded every 50 fs, which was used to analyze the evolution details of NONA in the pyrolysis process.

### 2.2 Rate constants analysis

The reaction rate of NONA in the decomposition process can be divided into three stages (endothermic stage, exothermic stage, and generation of gaseous products). This method has already been used to calculate the rate constants of TNT,^8^ CL-20,^12^ and HNS^14^ decomposition. The reaction rate of NONA at each stage can be described by the Arrhenius equation, shown below1
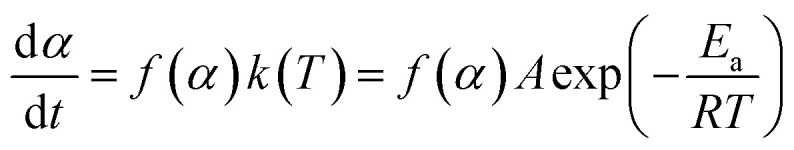
where *t*, *T*, *R* and *α* are time, temperature, gas constant and reaction progress, respectively. *A* is the pre-exponential factor, and *E*_a_ is the activation energy. The *A* and *E*_a_ can be obtained by linearly fitting. *f*(*α*) is the reaction model, and *k*(*T*) is the temperature-dependent rate constant. The linear form of Arrhenius law relates the rate constant to activation energy and temperature, calculated by the following equation2
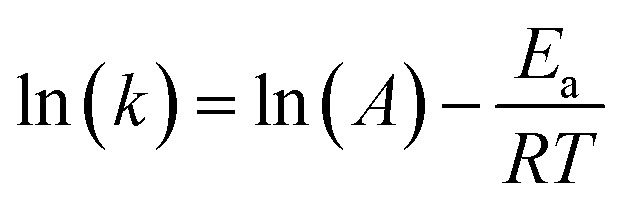


The three stages of the decomposition are:

(1) The initial decomposition reaction uses the first-order decay exponent *N*(*t*) to fit the reduction in the number of NONA molecules from *t*_0_ to *t*_max_. *t*_0_ and *t*_max_ are the times of the initial decomposition and the maximum potential energy, respectively.3
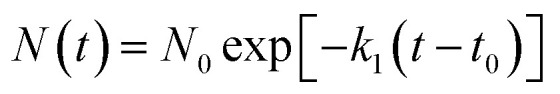
where *N*_0_ is the initial number of NONA molecules; *t*_0_ is the time at which NONA begins to decompose; *k*_1_ is the primary reaction rate.

(2) The intermediate decomposition reaction is an exothermic stage. The potential energy (PE) of the exothermic stage changes with time using the decay expression *U*(*t*)4
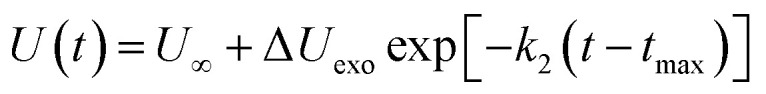
where Δ*U*_exo_ = *U*(*t*_max_) − *U*_∞_, *U*_∞_ is the equilibrium value of potential energy when *t* approaches infinity; *U*(*t*_max_) is the maximum potential energy, and *k*_2_ is the secondary reaction rate.

(3) The final product of the thermal decomposition of NONA supercell is fitted with time to obtain the final rate constant of product formation.5
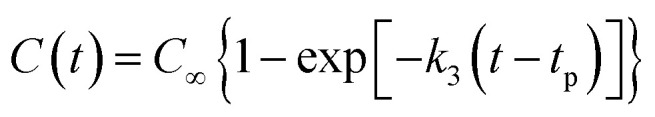
where *C*_∞_ is the asymptotic value when the amount of the product approaches equilibrium; *t*_p_ is the time at which the product begins to form; *k*_3_ is the final product formation rate.

## 3. Results and discussion

### 3.1 Validation of ReaxFF for NONA

**Fig. 2:**
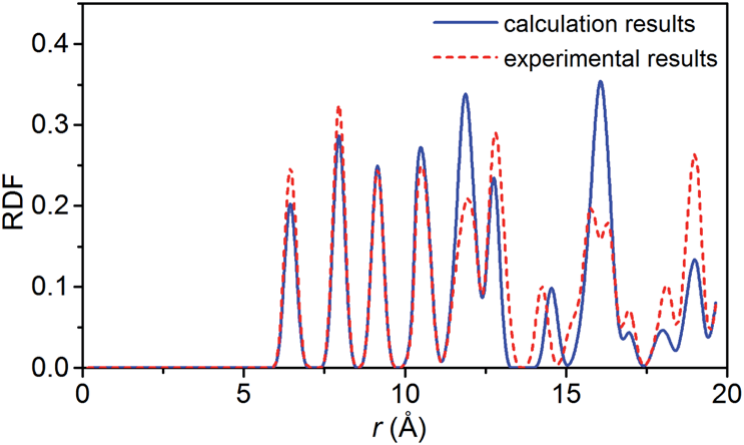
Comparison of the RDF from ReaxFF-lg (300 K, 0 atm) and from experiment for NONA.

To verify the feasibility of the ReaxFF-lg force field, we compared the lattice parameters, density and bond length of the relaxed NONA supercell at 300 K and 0 atm with the corresponding parameters obtained by X-ray single crystal diffraction.^6^ The calculation results are shown in Tables 2 and 3. By comparison, it was found that the unit cell parameters and density were in excellent agreement with the experimental values (error less than 1%). The average bond lengths of C–H, C–N, C–C, and N–O agree well with the experimental parameters. Among them, the larger difference occurred in the C–H and N–O bonds, which are 0.036 and 0.045 Å larger than the experimental values, respectively. The deviation between the calculated and experimental bond length of C–H and N–O is 3.87% and 3.69%. These errors can be ignored in simulation. Fig. 2 shows the comparison of the radial distribution function (RDF) of the NONA supercell obtained using ReaxFF-lg with experimental values. The RDF of the NONA molecular centroid at 300 K and 0 atm is close to the experimental values. The main peak positions calculated are 6.45, 7.95, 9.15, 12.75, 14.25 and 15.75 Å, whereas those obtained from the experimental data are 6.45, 7.95, 9.15, 12.75, 14.55 and 16.05 Å. Therefore, the ReaxFF-lg force field can be applied to the crystal structure simulation of NONA.

**Table 2:** Lattice parameters of NONA crystal

Methods	*a* (Å)	*b* (Å)	*c* (Å)	*α* = *β* = *γ* (°)	*ρ* (g cm^−1^)
Expt. (ref. 6)	16.822	11.957	23.097	90.0	1.82
ReaxFF-lg	16.850	11.977	23.136	90.0	1.81

**Table 3:** Comparison of bond lengths of NONA predicted *via* ReaxFF-lg (300 K, 0 atm) with the corresponding experimental parameters

Bonds	Bond length (Expt.)/Å	Bond length (ReaxFF-lg)/Å	Deviation/%
C–H	0.930	0.966	3.87
C–N	1.477	1.491	0.95
N–O	1.221	1.266	3.69
C–C (benzene rings)	1.404	1.440	2.56
C–C (sigma bond)	1.510	1.520	0.66

### 3.2 Evolution of the potential energy

**Fig. 3:**
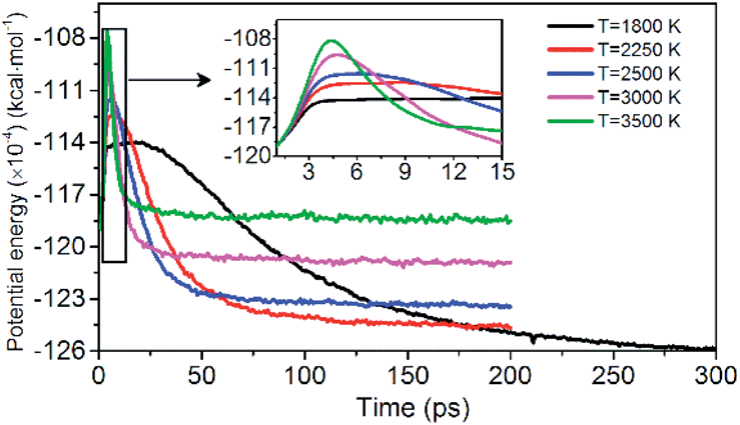
Variation of the PE with time at various temperatures. Black, red, blue, pink, and green represent 1800, 2250, 2500, 3000, and 3500 K, respectively.

Evolution of PE is shown in Fig. 3. The variation of potential energy for the system is clearly divided into endothermic (seen in insets of Fig. 3) and exothermic stages. At the initial pyrolysis, the system quickly absorbs heat to increase the potential energy of the system. Subsequent reactions produce a series of intermediate and final products and release a large amount of heat, thus a significant decrease in system potential energy. The change of PE of each system is closely related to the chemical bond cleavage and formation in thermal decomposition reaction. The higher the temperature, the shorter the time required for the system to reach the dynamic equilibrium of the chemical reaction.

### 3.3 Evolution of the chemical species

**Fig. 4:**
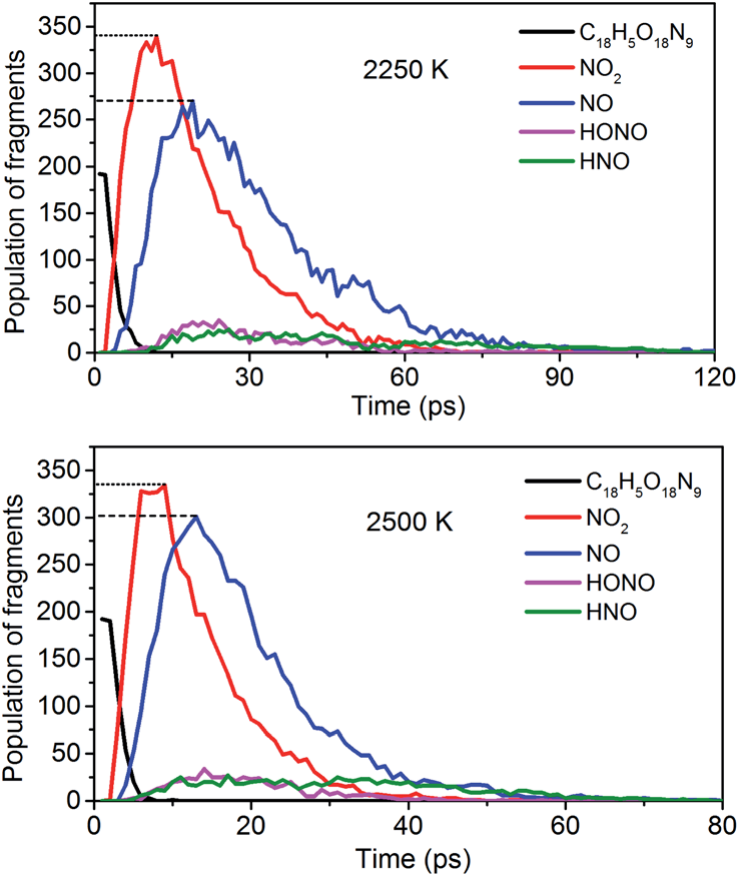
Evolution with temperature/time of NONA (C_18_H_5_O_18_N_9_), NO_2_, NO, HONO, and HNO at 2250 and 2500 K. The dotted line represents the maximum values of NO_2_ and NO.

**Fig. 5:**
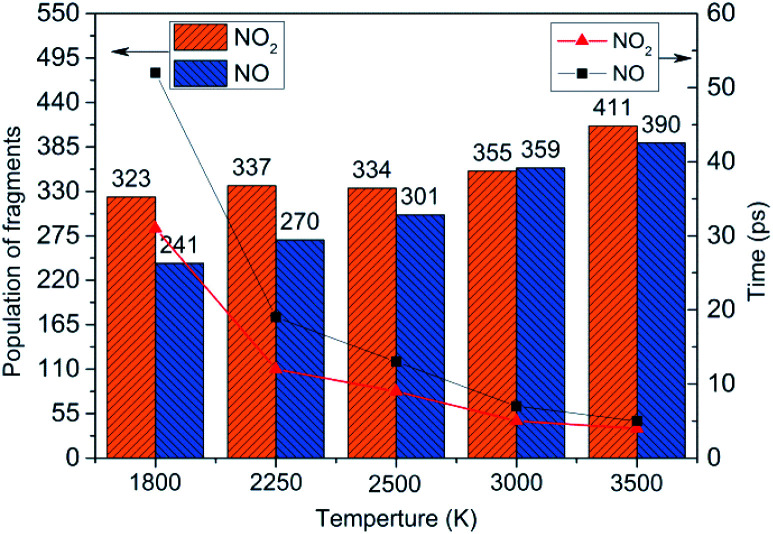
Peak value (column chart) and corresponding time (line chart) of NO_2_ and NO molecules at various temperatures.

To obtain the decomposition process of NONA, a series of C++ scripts were used to obtain the fragment statistics during the decomposition process of NONA. Fig. 4 shows the change of molecular fragments with time at 2250 and 2500 K. NONA molecules have been decomposed at 11.64 ps and 7.13 ps, respectively. At 2250 K, the initial reactions generate NO_2_ and NO occur at 1.96 ps and 3.54 ps, respectively, whereas at 2500 K, the initial generation times of these two species are reduced to 0.87 ps and 3.03 ps. In this respect, the reaction is accelerated by increasing temperature. NO appears slowly after NO_2_, indicating that NO_2_ generation is more likely to occur. The simulations reveal that two possible initial decomposition processes: (i) N–N homolysis and (ii) nitro–nitrite (NO_2_ → ONO) isomerization followed by the NO fission. The number of the abundance species NO_2_ and NO at five temperatures were summarized in Fig. 5 as time goes on the initial stage. More NO_2_ and NO are produced as temperature increases, while the difference in the maximum values of NO_2_ and NO is decreasing. Especially at 3000 K, the maximum value of NO exceeds that of NO_2_, indicating that the rearrangement of C–NO_2_ moiety to C–ONO followed by O–NO homolysis is a thermodynamically more favorable approach. In addition, the concerted HONO elimination is deemed to preferentially occur in the condensed phase energetic materials.^9,16–19^ However, it should be noted that the HONO eliminations is less favorable for the initial decomposition process of NONA. The decomposed products of NO_2_, NO and HONO reach their peak values at 9.15, 13.54 and 14.91 ps, respectively, corresponding to the species numbers of 334, 301 and 34. The amount of HONO generated is much smaller than those of NO_2_ and NO (as shown in Fig. 4). Furman *et al.*^9^ also proposed that the first pathway of intermolecular H-transfer is possible only if H atoms are available on the ring substituents (*i.e.*, –CH_3_, –NH_2_), since the cleavage of a benzene ring bound hydrogen requires much more energy. Hence, for NONA with only nitro substituents, the intermolecular H-transfer is not favorable to participate in the HONO eliminations since the numbers of HONO and HNO are so small.

### 3.4 Subsequent decomposition pathway

**Fig. 6:**
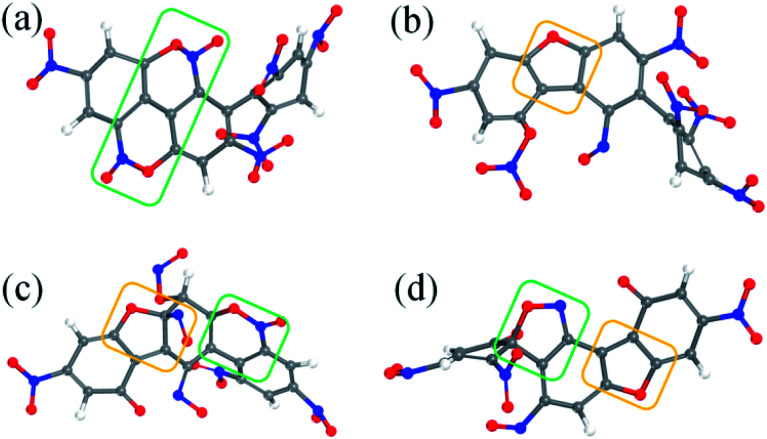
Snapshot of a series of annulation. The C, H, O, and N atoms are represented by gray, white, red, and blue balls, respectively. Four intermediates are denoted by (a–d). The generated five- and six-membered rings are in yellow and green boxes, respectively.

**Fig. 7:**
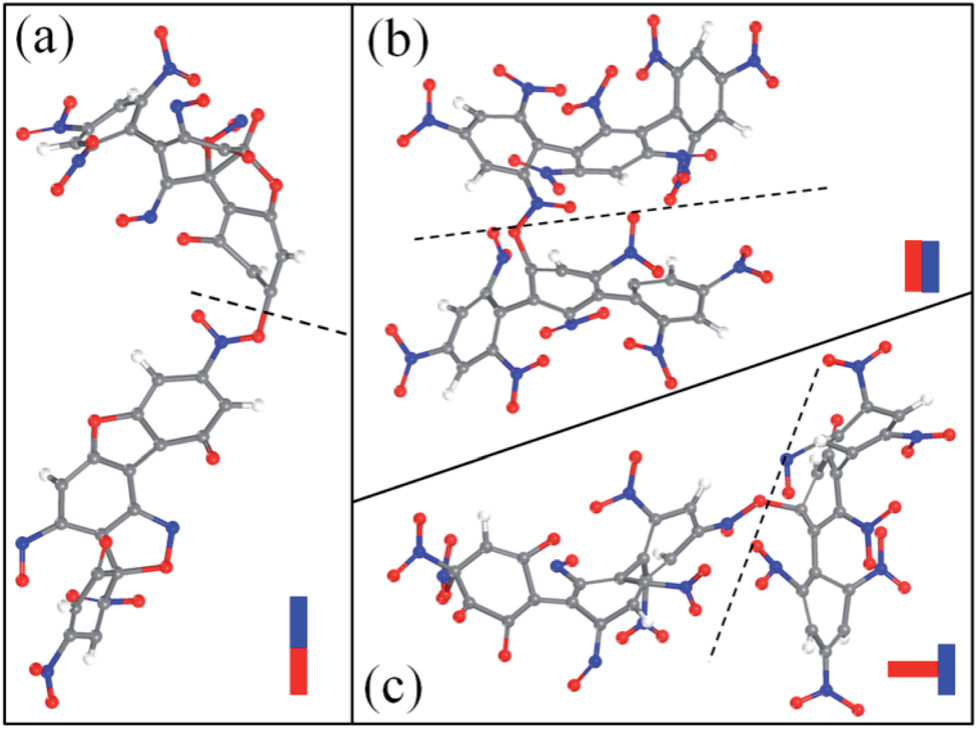
Snapshot of the bimolecular NONA. The docking modes are classified into three kinds: (a) head to head, (b) side to side and (c) side to head. The C, H, O, and N atoms are represented by gray, white, red, and blue balls, respectively.

After the homolysis of NO_2_ and NO, the simulation results show that there are macromolecular fragment products containing C_18_ and C_36_. Stability of terphenyl radicals might be responsible for their detection in the condensed phase. The cyclization reaction of terphenyl derivative occurs through the formation of N–O–C and O–C between the O and NO on the benzene ring and C radical in adjacent benzene ring (Fig. 6). The five- and six-membered rings are the derivatives of oxole and benzoxazine derivatives. In addition, the bimolecular mechanisms, especially for condensed phase, are a crucial decomposition process. However, most of the current researches are focusing on H transfer between bimolecules.^20,21^Fig. 7 shows docking modes for the bimolecular of NONA. Bimolecular is linked together by a C atom in one terphenyl derivatives removed from NO_2_ group and an O atom of another terphenyl derivative.

**Fig. 8:**
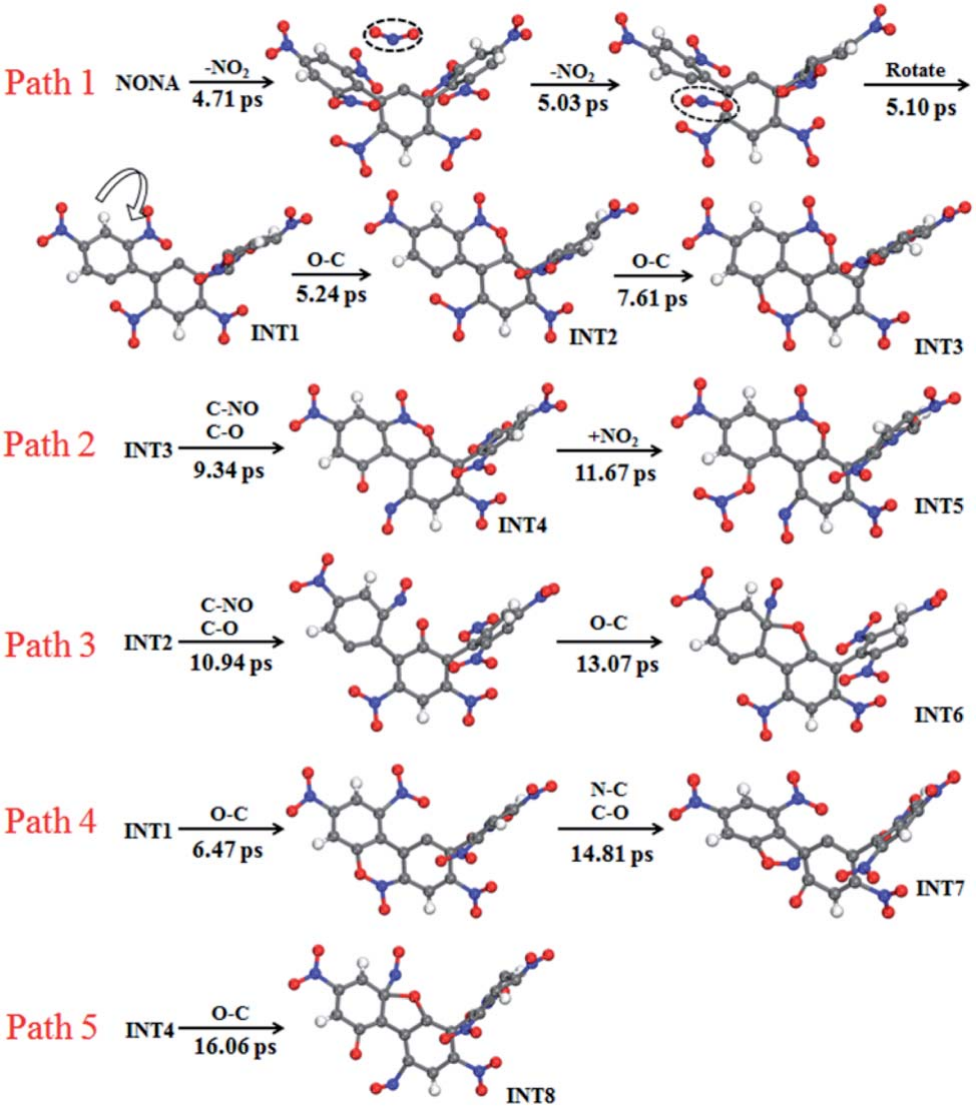
Reaction process of initial decompositions of NONA. The C, H, O, and N atoms are in gray, white, red, and blue, respectively. The partially decomposed NONA molecules are represented by INT1–INT8.

The mechanisms of five initial reactions are illustrated in Fig. 8. Each path is independent in the analysis of reaction mechanism. (1) Path 1: the initiation of thermal decomposition of NONA occurs through C–NO_2_ bond cleavage in intermediate benzene ring (*t* = 4.71 ps), leading to the formation of NO_2_. Then the another C–NO_2_ bond in side benzene ring of NONA breaks immediately after first NO_2_ dissociation (*t* = 5.03 ps), leading to the formation of the carbon radical on each benzene ring. These benzene rings fuse with its neighbor nitro groups after the twist of C–C single bond (*t* = 5.10 ps). The planes of the two benzene rings approach parallel, and then the O atoms in the nitro group and the C of the adjacent benzene ring form a C–O bond (5.24 ps). A similar reaction process occurs at 7.61 ps, and finally forms a fused ring compound (INT3). (2) Path 2: the N–O in benzoxazine structure breaks leading to the formation of C–O and C–NO (9.34 ps). The CO bonding with external NO_2_ radical to form nitrate ester C–ONO_2_ (11.67 ps). (3) Path 3: the INT2 structure undergoes N–O bond cleavage similar to the first step of Path 2 (10.94 ps). Then the attraction between the O and one C of the C–NO leads to the formation of C–O bonds at 13.07 ps. (4) Path 4: the formation of C–O bonds of the INT1 structure occurs at 6.47 ps. The C belonging to one benzene ring attracts the N belonging to –NO in an adjacent benzene ring, forming C–N bonds (14.81 ps). (5) Path 5: the mechanisms for O–C bond of INT4 structure formation are similar to the second step of Path 3 (16.06 ps).

### 3.5 Evolution of the gaseous and cluster products

**Fig. 9:**
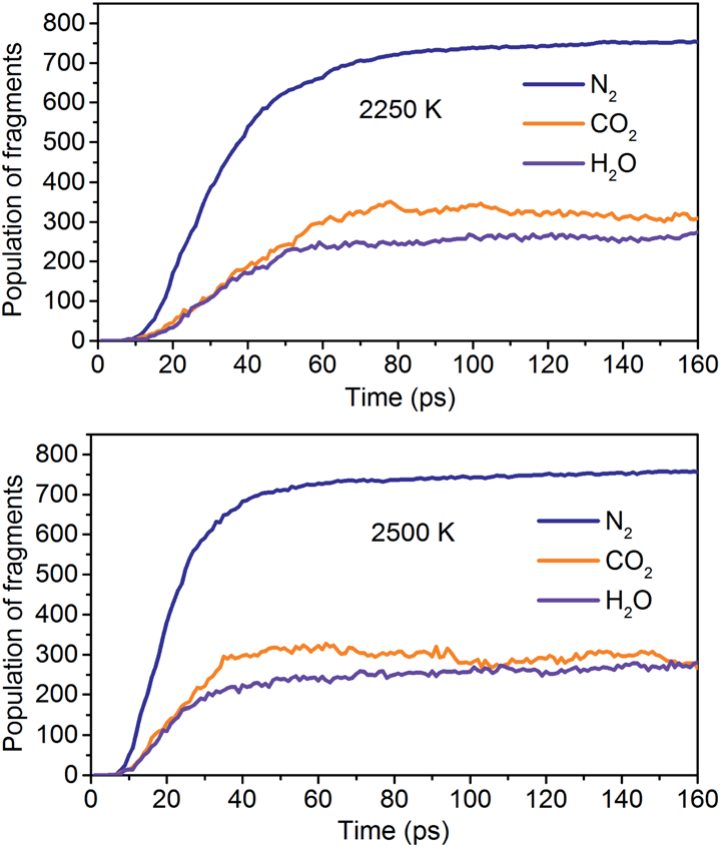
Evolution of the final products with time at 2250 K and 2500 K.

**Fig. 10:**
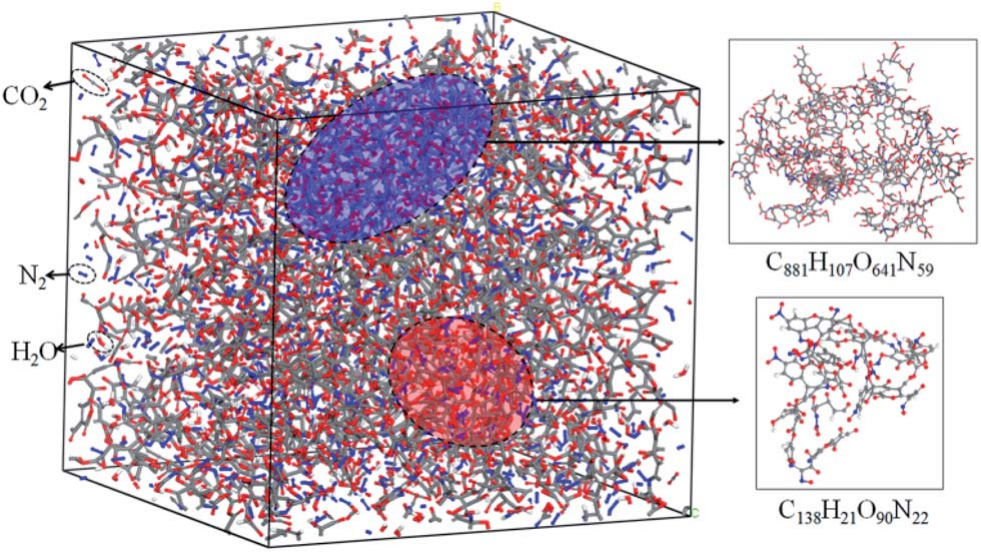
Snapshot of an equilibrium configuration at 200 ps and 2250 K. Final structures of N_2_, H_2_O, CO_2_, and large cluster formed in NONA.

The simulation results show that the final products of NONA decomposition are mainly N_2_, CO_2_ and H_2_O. Here, Fig. 9 shows the evolution of the final products with time at 2250 and 2500 K. The final products are produced continuously, and their amounts are nearly equilibrium at 180 ps. The order of the amounts of the final products are N_2_ > CO_2_ ≈ H_2_O at 180 ps. In addition to forming gaseous products, some clusters come into being in thermal decomposition of NONA. Fig. 10 shows a snapshot of the NONA supercell at 200 ps for 2250 K, in which the formation of both small and large products, such as N_2_, CO_2_, H_2_O and large clusters, can be observed. As the –NO_2_ and –NO bonds cleavage, the C-containing groups continue to polymerize to form large molecular clusters, which lead to a rapid increase in the molar mass of the carbon-containing clusters, followed by secondary chemical reactions. Large clusters are in a dynamic equilibrium by the cleavage of various small moieties and the polymerization of small clusters. The appearance of clusters is an important aspect to understand the properties of energetic materials. Experimental and theoretical researches have shown that carbon-rich explosives can form larger carbon-containing clusters in detonation process.^22–28^

### 3.6 Diffusion analysis

**Fig. 11:**
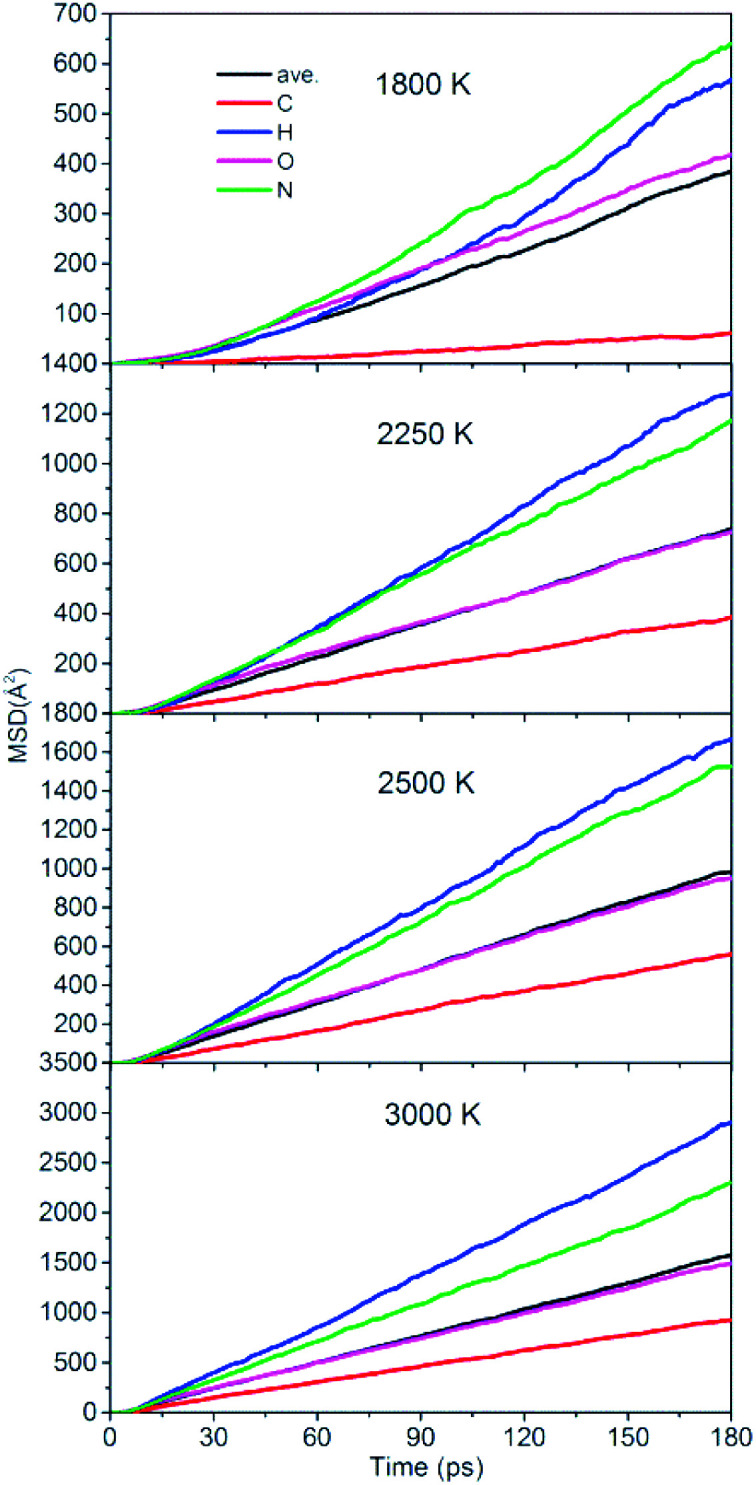
Mean squared displacement of C/H/O/N atoms of NONA at 1800, 2250, 2500, and 3000 K.

To quantitatively describe the interactions between the NONA molecules, the three-dimensional diffusion coefficients for all atoms can be calculated as the derivative of its mean square displacement (MSD) at four temperatures (Fig. 11). At 1800 K, the C, H, O, and N atoms are estimated from the slope of the MSD to be 3.38 × 10^−9^ m^2^ s^−1^, 3.13 × 10^−9^ m^2^ s^−1^, 2.31 × 10^−9^ m^2^ s^−1^, and 3.53 × 10^−9^ m^2^ s^−1^, respectively. The order of the MSD of other four atoms were N > H > O > C, which is not consistent with the order of atom mass O > N > C > H. This indicates that there are other factors that affect the MSD. On the one hand, N belongs to NO_2_ and participates in more reactions, whereas the amount of HONO and HNO are significantly less in the initial pyrolysis reaction. It is found that the intermediate of NONA retains the H atom in Fig. 6 and 7. On the other hand, a large amount of C atoms participate in the formation of clusters and make its diffusion more difficult. As the temperature increases, the diffusion rate of all atoms increases, and the diffusion rate of H atoms gradually exceeds that of N atoms. This indicates that the temperature has a significant acceleration on the diffusion coefficient of H atoms.

### 3.7 Thermal decomposition of evolution under two densities

**Fig. 12:**
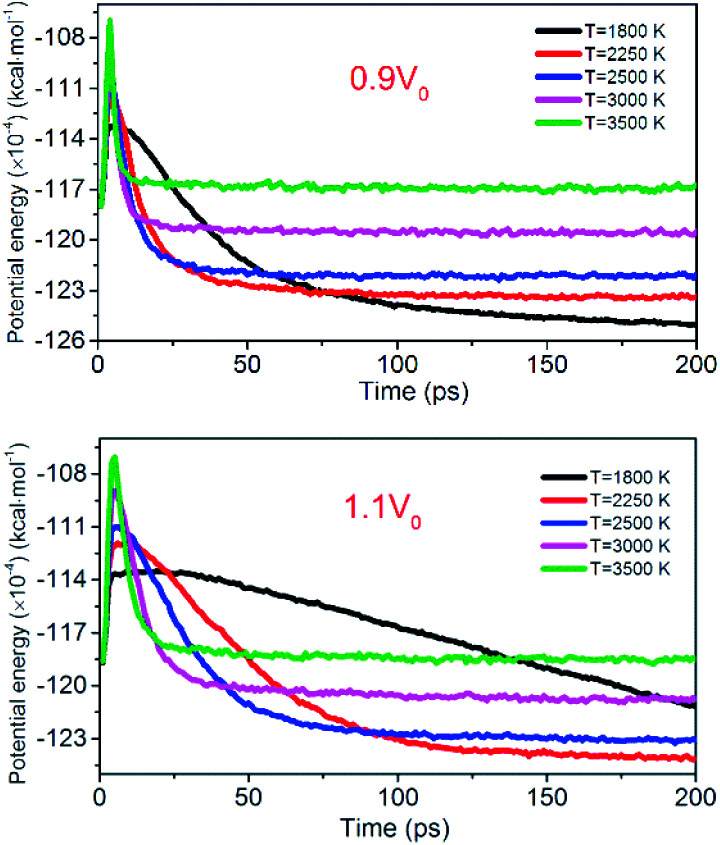
Variation of the PE with temperature/time at 0.9*V*_0_ and 1.1*V*_0_. Black, red, blue, pink, and green represent 1800, 2250, 2500, 3000, and 3500 K, respectively.

Fig. 12 shows the evolution of PE for the system of 0.9*V*_0_ and 1.1*V*_0_. When the volume of NONA supercells is reduced, and the corresponding atomic spacing and molecular spacing are also reduced. The equilibrium distance between molecules is reduced, and the repulsive force between molecules is dominant. On the contrary, the increase in the volume of NONA supercell makes the molecule and the atom appear attractive. The curve of PE for the different density of NONA shows an initial sharp increase followed by a slow decrease with time. The system of 0.9*V*_0_, *V*_0_ and 1.1*V*_0_ have a *t*_max_ of 4.16, 4.87 and 5.59 ps at 3500 K, respectively, indicating that the increases in the volume of NONA supercells cause the endothermic phase to be prolonged. We subtract *t*_max_ from the reach equilibrium time of *t*_eq_. The time required for the 0.9*V*_0_, *V*_0_, and 1.1*V*_0_ systems to reach equilibrium during the exothermic phase were 12.15, 18.04, and 23.73 ps, respectively. This indicates that the increases in the volume of NONA supercells also prolong the exothermal time.

**Fig. 13:**
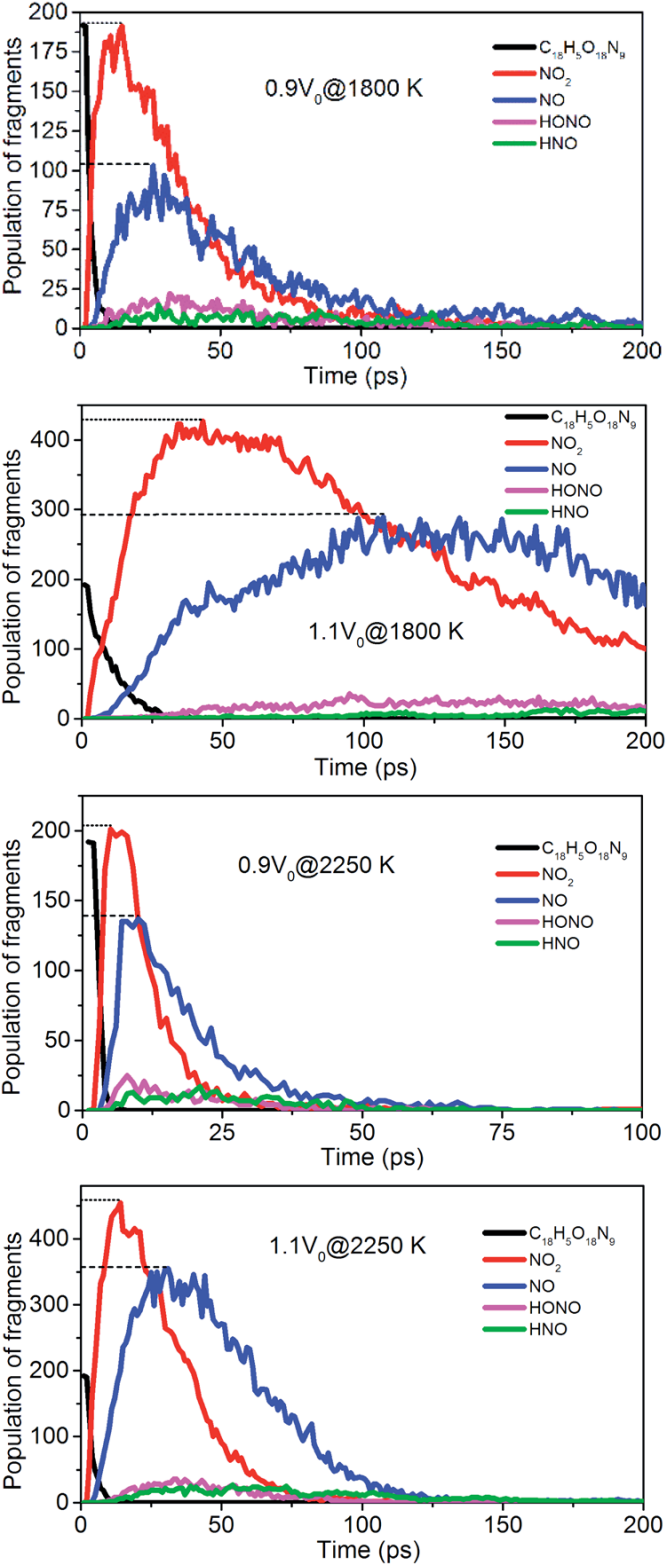
Evolution with time of NONA (C_18_H_5_O_18_N_9_), NO_2_, NO, HONO, and HNO for 0.9*V*_0_@1800 K, 1.1*V*_0_@1800 K, 0.9*V*_0_@2250 K and 1.1*V*_0_@2250 K. The dotted line represents the maximum values of NO_2_ and NO.

To evaluate the degree of NONA decomposition for volumetrically expanded and compressed supercells of 1.1*V*_0_ and 0.9*V*_0_ at 1800 and 2250 K, the variations of remaining HMX and major decomposition products with time are showed in Fig. 13. These ReaxFF-lg MD simulations elaborate that the N–N homolysis and nitro–nitrite (NO_2_ → ONO) isomerization followed by the NO fission dominate the initial stage of NONA dissociation in the system of 1.1*V*_0_ and 0.9*V*_0_. The decomposition schemes for NONA in 1.1*V*_0_ and 0.9*V*_0_ are similar to that for *V*_0_ system except the amount and formation time of NO_2_ and NO.

At 2250 K, NO_2_ begins to appear at 5.14, 12.83, and 14.11 ps for the system of 0.9*V*_0_, *V*_0_, and 1.1*V*_0_, respectively, and the corresponding amount of NO_2_ were 201, 339, and 454. NO_2_ increases more quickly with the increase in density. Similarly, NO occurs at about 10.02, 19.24, and 30.63 ps for the system of 0.9*V*_0_, *V*_0_, and 1.1*V*_0_, respectively, and the corresponding amount of NO were 137, 270 and 354. Density has the same effect on the amount of NO.

**Fig. 14:**
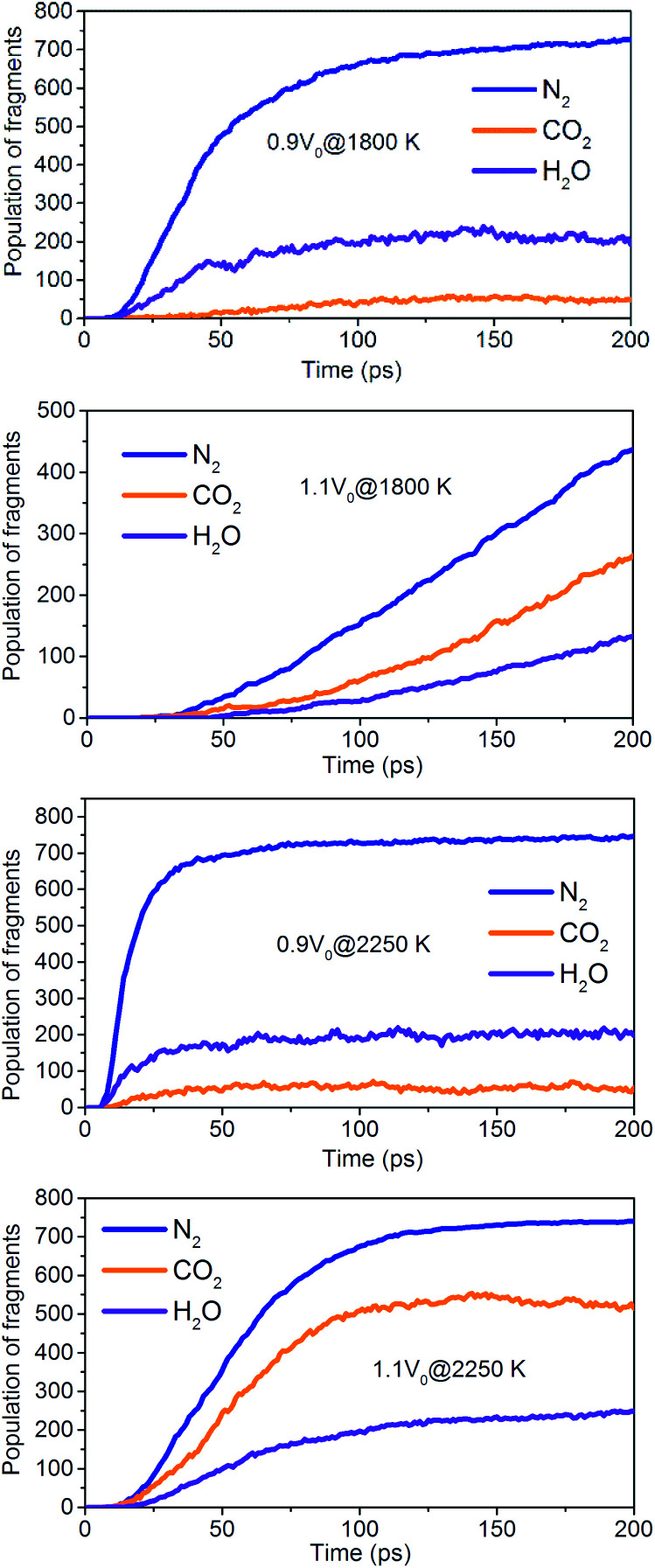
Evolution with temperature/time of the final products for 0.9*V*_0_@1800 K, 1.1*V*_0_@1800 K, 0.9*V*_0_@2250 K and 1.1*V*_0_@2250 K.

The onset of various secondary exothermic reactions leads later to the formation of many final products, such as N_2_, CO_2_, and H_2_O (Fig. 14). The equilibrium time required for N_2_ and H_2_O is shortened to a low-density system of NONA, while the amount of N_2_ and H_2_O fragments remains stable. However, the formation of CO_2_ is favored at a low-density system of NONA. The low-density system of NONA is not conducive to the formation of C clusters, and more C detaches from clusters and forms CO_2_.

### 3.8 Reaction kinetic parameter analysis

To understand the effects of temperature and pressure for the decomposition process of NONA, the reaction kinetic parameters were employed and evaluated for the three different reaction stages.

**Fig. 15:**
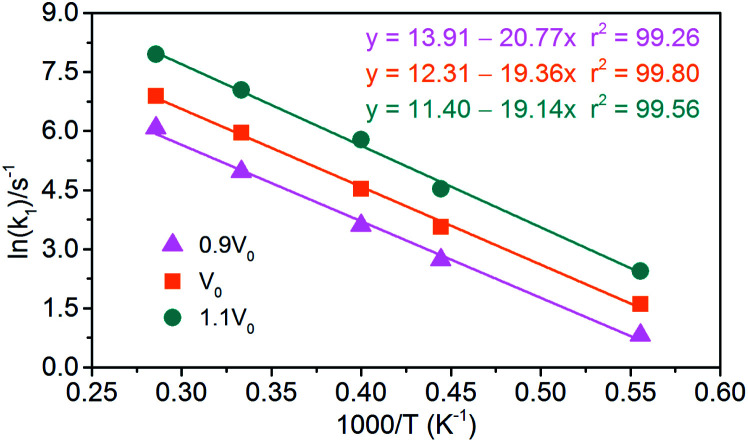
Logarithm of NONA initial decomposition rate constant ln(*k*_1_) against (1/*T*) in range of 1800–3500 K. The solid point and solid line represent calculated and linearly fitted values for three density systems. Orange, magenta, and dark cyan represent the systems of 0.9*V*_0_, *V*_0_, and 1.1*V*_0_, respectively.

(1) Endothermic stage: the system costs about 1.4 ps to heat to the target temperature. Hence, for endothermic stage, we should study the reduction process of NONA from 1.4 ps to *t*_max_. Table 4 lists the values of *t*_max_ for the system of *V*_0_, 0.9*V*_0_ and 1.1*V*_0_. Using eqn (3), we obtain the initial decomposition rate constants (*k*_1_) at 1800, 2250, 2500, 3000, and 3500 K of the initial decomposition of NONA with different crystal density. The *k*_1_ becomes larger with increasing crystal density and/or temperature, indicating that density and temperature can accelerate the decomposition of NONA. The pre-exponential factors and activation energies can be obtained by establishing a linearly fitted eqn (2) (Fig. 15). These values are summarized in Table 5. The fitted activation energy in the simulation is the apparent activation energy for overall reactions. The *E*_a_ values (38–41 kcal mol^−1^) are lower than the reported experimental values (51.14 kcal mol^−1^) because some highly reactive radicals and intermediates take part in the reactions in addition to NONA decomposition when the temperatures are in the range of 1800 K to 3500 K.^15^

**Table 4:** *t*_max_ (ps) at various temperature

Temperature (K)	*t*_max, 0.9*V*_0__	*t*_max, *V*_0__	*t*_max, 1.1*V*_0__
1800	7.06	18.45	29.44
2250	5.84	7.37	9.54
2500	5.17	6.75	8.45
3000	4.90	5.41	6.28
3500	3.87	4.16	5.59

**Table 5:** Pre-exponential factors and activation energies for endothermic and exothermic stages of NONA thermal decomposition

Stage	Model	ln *A* (s^−1^)	*E*_a_ (kcal mol^−1^)
Zeman *et al.*^5^		35.01	56.48
Keshavarz *et al.*^15^	—	—	51.14
Endothermic stage	0.9*V*_0_	13.91	41.27
	*V*_0_	12.31	38.47
	1.1*V*_0_	11.40	38.03
Exothermic stage	0.9*V*_0_	17.17	49.82
	*V*_0_	18.28	49.64
	1.1*V*_0_	19.27	49.34

**Fig. 16:**
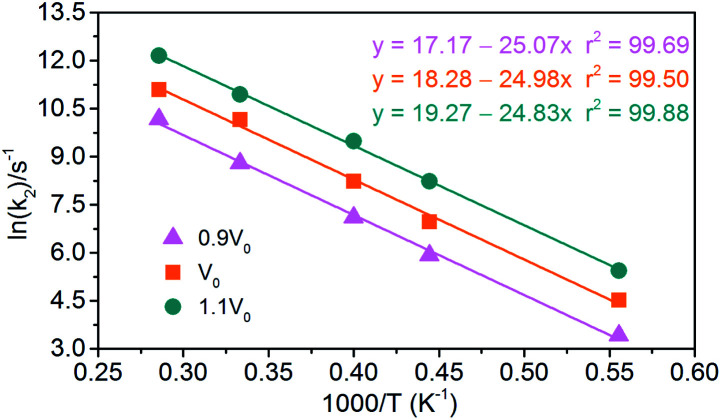
Logarithm of NONA second decomposition rate constant ln(*k*_2_) against (1/*T*) in the 1800–3500 K range. The solid point and solid line represent calculated values and linear fitting for three density systems. Orange, magenta and dark cyan represent the systems of 0.9*V*_0_, *V*_0_ and 1.1*V*_0_, respectively.

**Fig. 17:**
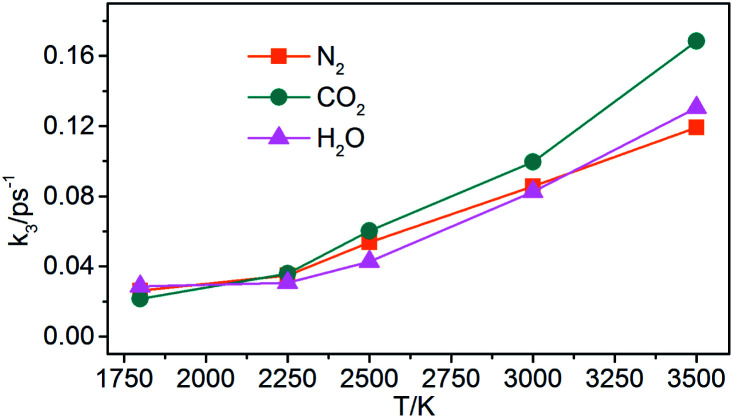
The reaction rate of the final products (*k*_3_) *vs.* the temperature for the system of *V*_0_. Orange, magenta and dark cyan represent N_2_, CO_2_ and H_2_O, respectively.

(2) Exothermic stage: the second decomposition rate constants (*k*_2_) are obtained by fitting the attenuation of PE curve with eqn (4). The results of linear fitting, as seen in Fig. 16, are extracted with eqn (2). The data of pre-exponential factors and activation energies for various systems is presented in Table 5. A higher temperature leads to a higher secondary decomposition rate, whereas the greater density results in smaller activation energy. The calculated activation energies agree well with the experimental data from Russian manometric (isothermal) method.^5^

(3) Generation of gaseous products: the gaseous products are mainly N_2_, CO_2_, and H_2_O in NONA decomposition process. The rate constants (*k*_3_) of the gaseous products are obtained by fitting these data of gaseous products into eqn (5). As shown in Fig. 17, the *k*_3_ is increased from 1800 to 3500 K, and CO_2_ with faster formation rate is observed at the NONA decomposition stage as the temperature increases.

## 4. Conclusions

We simulated and analyzed the thermal decomposition reaction of NONA crystals at different temperatures by using ReaxFF-lg reactive force field. The primary initial reaction pathways are obtained by analyzing the evolution of the chemical species. We propose that two distinct initial decomposition mechanisms are the homolytic cleavage of C–NO_2_ bond and the rearrangement-homolysis pathway of C–NO_2_ → C–ONO followed by the NO fission. The fused ring clusters and bimolecules were found in the subsequent decomposition of NONA. The latter is a thermodynamically more favorable pathway than the C–NO_2_ homolysis at high temperature. The identification analysis of final products showed that the gaseous products were CO_2_, N_2_, and H_2_O. The amount of CO_2_ will be more favorable energetically for the system at high temperature or low density, and the clusters are a favorable growth pathway in low temperatures, and this process was further demonstrated by the analysis of diffusion coefficients. Crystal density has a significant acceleration on the decomposition of NONA through increasing reaction kinetic parameter (*k*_1,2_) and reducing activation barriers.

## Conflicts of interest

There are no conflicts to declare.

## Supplementary Material
